# Seventh Annual Announcement of the Medical Department of the Iowa University

**Published:** 1856-08

**Authors:** 


### SEVENTH ANNUAL ANNOUNCEMENT

OF THE

### College of Physicians and Surgeons

OF THE

### IOWA STATE UNIVERSITY.

### BOARD OF TRUSTEES.

HON. JAMES D. EADS, PRES'T, OF THE BOARD, EX OFFICIO.
HON. ANSON HART, SECRETARY, Iowa City.
HIS EXCEL. JAMES W. GRIMES, TRUSTEE, EX OFFICIO.

HON. GEO. W. McCLEARY, Iowa City.
" ANSON HART, " "
" E. C. LYON, " "
" JAMES H. GOWER, " "
" G. D. PALMER, " "
} Terms expire in 1857

" EDW. CONNELLY, " "

" H. W. LATHROP, " "

" M. J. MORSMAN, " "

" J. W. RANKIN, Keokuk.

} Terms expire in 1859.

" P. L. LAKE, Bellevue.
" LAURIN DEWEY, Mount Pleasant.
" THOS. FARMER, M'Kissack's Grove.
" E. C. BIDWELL, Quasqueton.
" AMOS WITTER, Davenport.
} Terms expire in 1861.

HON. LINCOLN CLARK, (to fill vacancy,) Dubuque, term expires in 1859.

### CURATORS.

HON. GEORGE G. WRIGHT, Chief Justice of the Supreme Court.
" W. S. WOODWARD, Associate " " "
" L. D. STOCKTON, " " " "

HON. R. P. LOWE, Judge I District.
" THOS. S. WILSON, Judge II District.
" H. B. HENDERSHOT, " III "
" WM. SMITH, " IV "
" J. C. McFARLAND, " V "
" A. A. BRADFORD, " VI "
" SAM'L H. RIDLLE, " VII "
" W. H. TUTHILL, " VIII "
" J. S. TOWNSHEND, " IX "

KEOKUK, IOWA:

1856.

### ANNOUNCEMENT

FOR THE

### SESSION OF 1856-'57.

---

AMOS DEAN, L. L. D., PRESIDENT OF THE UNIVERSITY.

---

**MEDICAL FACULTY,**

D. L. McGUGIN, M. D.,
Professor of Physiology, Pathology, Microscopy, and President of the Medical Faculty.

FREEMAN KNOWLES, M. D.
Professor of Theory and Practice of Medicine.

J. C. HUGHES, M. D.,
Professor of Surgery and Treasurer of the Faculty.

WELLS R. MARSH, M. D.,
Professor of Materia Medica, Chemistry and Agricultural Chemistry.

JOHN R. ALLEN, M. D.,
Professor of Obstetrics and Diseases of Women and Children, and Dean of the Faculty.

DANIEL MEEKER, M. D.,
Professor of Anatomy.

JOHN J. PAGE, M. D.
Demonstrator of Anatomy.

---

A. M. CARPENTER, M. D.,
Prosector to the Chair of Surgery.

J. WARD DAVIDSON, M. D.,
Prosector to the Chair of Anatomy.

J. R. ALLEN, M. D., DEAN.

---

THE Faculty of the Medical Department of the Iowa University teel that they may assure the profession, and the public, that the prospects of the School are, in every way, encouraging —the last class having been a large increase upon any previous one.

Taking into consideration that the location of this Department of the University, at Keokuk, is among the most eligible in the northwest, being at the head of uninterrupted navigation on the Mississippi, two hundred miles north of St. Louis, and three hundred miles south-west of Chicago, in a rapidly growing city, already sufficiently large to secure the usual facilities for demonstration, of easy access from any part of our own and neighboring States, the Faculty are confident that the present success is the certain promise of future increased and permanent prosperity.

A review of the history of the Institution, which may be had in the Catalogue of the University, will show that the efforts to build up this department of our State Institution have thus far equalled, even the highest hopes of its friends. Encouraged by such results, the Faculty are still more determined to leave no proper means untried to realize the hopes which previous successes incite.

In addition to personal efforts, the means of illustration in each department are being from time to time augmented by such apparatus, plates, &c., as the means at their command afford.

The Faculty, composed principally of western men, engaged in the active duties of the profession, take pains to inculcate, both in doctrine and practice, such principles as apply to the peculiarities of our region of country.

The object steadily aimed at by the Faculty, is, by means of clear and explicit Lectures, and by frequent reviews and examinations, to make the course of instruction thorough, rather than brilliant; definite and permanent, rather than captivating and superficial. It is desired that the student may feel that he has been a permanent gainer by his attendance, and that he has gone *through* instead of *over*, a systematic course of Medical instruction.

SCHOLARSHIPS.

In order to disseminate, as widely as possible, the beneficial influence of this Institution, the Trustees of the Department have thought proper to establish State Scholarships, a limited number of certificates of which are lodged in the hands of the principal State officers, to be conferred, by them, upon such worthy practitioners, or students of Medicine, in their several districts as may desire the benefits conferred. These certificates entitle the holder of the tickets to a full course of Lectures, upon the payment to the Dean, at the beginning of the course, of $15.

These privileges were originally intended exclusively for our own State, but as applications are occurring from neighboring States, the Trustees reserve the right to bestow upon such applicants similar advantages, until the wants of our own State demand the exclusive benefits of this arrangement.

Those wishing to secure for themselves the advantages of these scholarships should apply to the Dean of the Faculty or to the Hon. Anson Hart, Secretary of the Board of Trustees.

To all others, the fees will be as heretofore.

UNIVERSITY HOSPITAL.

This humane institution, on the part of the city, was constructed in immediate connection with the College buildings, in order to derive the benefits of the care of the Faculty, and at the same time to furnish to the classes an opportunity for clinical instruction. It will be so arranged, that the students may enjoy, equally, all the advantages to be derived from it. Frequent visits will be made to the bedside of the patient, and cases that admit of removal, will be brought to the lecture room, and explained to the class.

In practical anatomy the advantages are ample. The supply of material is abundant, and every student is required to dissect one session. The personal supervision of the Professor of Anatomy, and the attendance of the Demonstrator, will afford every opportunity for improvement.

There is, in the College buildings, a museum, already valuable, and yearly becoming more so. It includes numerous valuable and accurate botanical plates, a great variety of casts and models, including many wax preparations, a splendid suit of Materia Medica, abundant dry and wet anatomical preparations and morbid specimens and many interesting illustrations of natural history, Geology, &c.

The Faculty take pleasure in announcing that a brief course of lectures upon Medical Jurisprudence, may be hoped for from Amos Dean, President of the University, during the coming session.

A course of twenty evening lectures on Agricultural Chemistry will be delivered, commencing on Monday, January 5th, and it is hoped that the farmers of the state will avail themselves of the opporiunity thus offered for their benefit. There will be no fee for this course, those wishing to attend being expected to pay the matriculation fee of three dollars.

To increase the number of surgical operations, and induce patients from a distance to present themselves, all necessary arrangements have been made for their accommodation by the Professor of Surgery.

A large number of minor and capital surgical operations were performed before the last class, among the latter of which were three of Lithotomy.

*All Surgical operations before the class are performed gratuitously.*

### COURSE OF LECTURES.

The regular Course of Lectures will commence on Thursday, the 30th day ot October. Introductory by Prof. W. R. Marsh.

Six lectures will be delivered daily on the various branches of Medical Science, and the session will continue four months, as heretofore. The annual Graduation exercises will be held, and the degrees conferred, at the close of the term. Every student will be required, within two weeks after the opening of the Session, to take his tickets and pay the regular fees.

The following are the requisites for the Diploma:

1st. The candidate must be twenty-one years of age—and present testimonials of a good moral character.

2nd. He must have attended two full courses of Medical Lectures; one of which must have been delivered in the Medical Department of the Iowa State University, or evidence of three years reputable practice will be considered equivalent to one course.

3d. The candidate must have studied Medicine three years, (including the course of Lectures) under the direction of a respectable medical practitioner.

4th. He must furnish an original Medical Thesis, (in his own handwriting, accompanied with his graduation fee,) which must be handed to the Dean, by 1st Day of February, and which shall, upon examination, prove satisfactory.

5th. He must, at the close of the term, pass an examination satisfactorily to the Faculty.

### FEES, &c., &c.

The fees for a full course of Lectures amount to $60, being $10 for each Professor; this will be adhered to, except in the case of Scholarships. The student may attend one or more of the courses as he may be disposed, and pay only for the lectures for which he enters.

The fee for the Diploma is $30. Matriculation ticket $3. The fee for admission to the dissecting rooms and demonstrations is $5.

Members of the profession from every part of the country, who are Graduates of Medicine, will, on presenting their Diploma to the Dean and paying a Marticulation fee, be admitted gratuitously to all the lectures..

Board can be obtained in the city at from $2 50 to $3 50. Students, upon arriving in the city, can obtain information as to good boarding houses, by calling at the office of the Dean, in Post Buildings, on 3d between Johnson and Main streets.

Medical Books may be purchased at the book stores in our city, on as good terms as in any western city.

All letters of inquiry may be addressed to

JNO. R. ALLEN, M. D., Keokuk,
*Dean of the Faculty.*

---

### BOOKS OF REFERENCE.

*Physiology and Pathology.*—Carpenter's, Kirk and Paget's Physiology, Williams' and Stille's Pathology.

*Theory and Practice.*—Wood's, Dunglingson's, Watson's Practice; Williams' Principles of Medicine.

*Surgery.*—Chelius, Miller's Principles and Practice, Perrie, Erricson, Liston, Pancoast; Druit.

*Materia Medica and Chemistry and Agricultural Chemistry.*—Pereira, Royle, United States Dispensatory, Stockhardt, Turner & Kayne's Chemistry, Leibig & Johnston's Agricultural Chemistjy.

*Obstetrics.*—Churchill, Meigs, Velpeau.

*Anatomy.*—Wilson's and Cruvilier's Anatomy, Smith and Horner's Atlas.

### MATRICULANTS FOR THE SESSION OF 1855-56.

| NEMES. | RESIDENCES. | PRECEPTORS. |
|---|---|---|
| Adamson, V. V. | Iowa. | Dr. Rodgers. |
| Adams, Geo. G. | Iowa. | " Morgan. |
| Ayres, D. | Iowa. | " Johnson. |
| Ayres, H. H. | Iowa. | Druggist. |
| Barber, S. | Iowa. | Dr. Barber. |
| Beerbower, J. | Iowa. | Dr. Norris. |
| Bishop, J W. | Illinois. | " Pale. |
| Bogen, W. L. | Ind. | Drs. Tilman & Mason. |
| Boude, J. K. | Ill. | Dr. Boude. |
| Boone, Henry | Iowa. | " D. Murry. |
| Brown, John J. | Mo. | Prof. Jeter. |
| Carpenter, H. | Iowa. | Dr. Carpenter. |
| Collins, J. B, | Ills: | Profs. M'Gugin & Allen |
| Cook, S. D. | Iowa. | Practitioner. |
| Collins, D. C. | Iowa. | |
| Cook, D. S. | Iowa. | Drs. Marsh & Irwin. |
| Coons, James N. | Mo. | Prof. Jeter. |
| Davidson, J. Ward | Ohio. | " Hughes. |
| Dunham, C. S., M. D. | Ills. | Practitioner. |
| Evans, A. J. | Iowa. | Dr. Miller. |
| Fletcher, R. J. | Ky., | Prof. Hughes. |
| Folmsbee, W. H. | Iowa. | Practitioner. |
| Gordon, J. | Iowa. | Dr. Gordon. |
| Gillett, L. | Ills. | " Carpenter. |
| Givens, W. H. | N. Y., | Prof. Hughes. |
| Githens, W. H., M. D. | Ills. | Practitioner. |
| Gilfillen, G. W. | Penn. | Dr. Swartz. |
| Griffith, M. Cassell, | Ills. | " Griffith. |
| Gray, Lafayette | Ills. | " Hall. |
| Hare, H. J. | Ills. | " Payne. |
| Harris, R. W. | Iowa. | " Ryland. |
| Henderson, James, | Ills. | Practitioner. |
| Henry, John | Mo. | " |
| Hamill, D. W. | Iowa. | Dr. Hamill. |
| Jeffries, P. C. | Iowa. | " Wood. |
| Kelley, James | Ills. | " M'Vay. |
| Kirlin, P. M. | Ills. | " Payne. |
| Kinkade, Wm. R. | Va. | Practitioner. |
| Ladd, J. A. | N. Y., | Dr. J. R. Leal. |
| Lynch, A. | Penn. | " Carpenter. |
| Leas, J. J. | Mo. | " Taylor. |
| La Force, James W, | Iowa. | Practitioner. |
| Martin, David R. | Iowa. | " |
| Mason, J. | Ills. | Dr. Mason. |
| Moore, Robert | Penn. | Prof. Hughes. |
| Murphy, Geo. | Mo. | Dr. Eadland. |
| Myler, J. M. | Ind. | Drs. Stewart & Moore. |
| McGinis, J. | Mo. | Dr. Hard. |
| McIntire, E. S. | Ind. | Drs. Debruler & Crooks |
| McKee, Robert, | Mo. | Prof. Hughes. |

| NAMES. | RESIDENCES. | PRECEPTORS. |
|---|---|---|
| McKinney, T. I. | Iowa. | Druggist. |
| Nelson, Geo. W. | Ills. | Dr. Nelson. |
| Newell, O. R. | Ills. | Drs. Young & Hard. |
| Norris, J. H., M. D. | Iowa. | Practitioner. |
| Nowottny, Jno. I. | Ohio. | Dr. Gordon. |
| Pallard, Geo. W. | Ills. | " Dunham. |
| Potter, Jno. W. | Ills. | " Mason. |
| Pringle, Henry L. | Iowa. | Practitioner. |
| Pyle, Elliott, | Iowa. | Dr. Atkinson. |
| Reed, F. B. | Iowa. | Practitioner. |
| Rhea, Lewis J, | Ills. | Profs. M'Gugin & Allen |
| Ritchie, Adelbert L. | Ills. | Dr. Richie. |
| Rosseau, D. H., M. D. | Iowa. | Practitioner. |
| Sayre, Jas. W. | Ky., | Prof. Hughes. |
| Solls, H. J. | Iowa. | Practitioner. |
| Shelton, W. A. | Iowa. | Dr. Allen. |
| Skinner, J. O., M. D. | Iowa. | Practitioner. |
| Stratton, F. M. | Ills. | Dr. Payne. |
| Stillman, C. B. | Ills. | " Arnold. |
| Spangenberg, D. | Germ'y. | " Page. |
| Streeter, E. C. | Ills. | Prof. Marsh. |
| Tollman, W. L. | Mo. | Dr. Faris. |
| Trimble, Jno. | Iowa. | " Green. |
| Turner, Luther, | Mo. | " Coons. |
| Van Nest, J. S. | Mo. | " Ellery. |
| Warford, F. M. | Iowa. | |
| Wade, W. H. | Ills. | " Dunham. |
| Williams, H. C. | Iowa. | " Van Paten. |
| Williams, T. J. | Ohio. | " Blazer. |
| White, J. A. H. | Mo. | Prof. Jeter. |
| Winans, H. N. | Ills. | Dr. Githens. |
| Wright, Geo. W. | Ills. | " Hill. |

### GRADUATES OF 1855–'56.

| NAME. | RESIDENCE. | SUBJECT OF THESIS. |
|---|---|---|
| V. V. ADAMSON, | Iowa, | Miasmus. |
| J. BEERBOWER, | Iowa, | Remote Causes of Nervous Diseases. |
| J. J. BROWN, | Mo., | Cerebral Apoplexy. |
| H. CARPENTER, | Iowa, | Circulation of the Blood. |
| J. W. DAVIDSON, | Iowa, | Cholera Infantum. |
| A. J. EVANS, | Iowa, | Malignant Cholera, |
| J. HENERY, | Mo., | Uterine Hemorrhage. |
| W. R. KINKADE, | Va., | Puerperal Convulsions. |
| J. W. LA FORCE, | Iowa, | Duties of Med. attendants in Natural Labor. |
| J. A. LADD, | N. Y., | Mania |
| ROBT. MOORE, | Iowa, | Changes in the Blood. |
| D. R. MARTIN, | Iowa, | Placenta Previa. |
| F. B. REED, | Iowa, | Physiology of Digestion. |
| L. J. RHEA, | Ills., | Glandular Secretion. |
| J. M. SAYERS, | Ky., | Hygienic Management of Children. |
| F. M. STRATTON, | Ky., | Dysentery. |
| C. B. STILLMAN, | Ills., | Effusion of Serum. |
| J. K. TRIMBLE, | Iowa, | Gonorrhea. |
| L. TURNER, | Mo., | Typhoid Fever, |
| J. S. VAN NEST, | Mo., | Etiology. |
| J. A. H. WHITE, | Mo., | Typhoid Fever. |
| F. M. WARFORD, | Iowa. | Calorification |
| T. J. WILLIAMS, | Ohio. | Tetanus. |

